# Eight Up-Coming Biotech Tools to Combat Climate Crisis

**DOI:** 10.3390/microorganisms11061514

**Published:** 2023-06-07

**Authors:** Werner Fuchs, Lydia Rachbauer, Simon K.-M. R. Rittmann, Günther Bochmann, Doris Ribitsch, Franziska Steger

**Affiliations:** 1 Department IFA-Tulln, Institute of Environmental Biotechnology, University of Natural Resources and Life Sciences, Vienna, Konrad-Lorenz-Strasse 20, 3430 Tulln, Austria; 2 Lawrence Berkeley National Laboratory, Deconstruction Division at the Joint Bioenergy Institute, 5885 Hollis Street, Emeryville, CA 94608, USA; 3 Archaea Physiology & Biotechnology Group, Department of Functional and Evolutionary Ecology, Universität Wien, Djerassiplatz 1, 1030 Wien, Austria; 4 ACIB—Austrian Centre of Industrial Biotechnology, Krenngasse 37, 8010 Graz, Austria

**Keywords:** climate change, biotechnology, Wood–Ljungdahl pathway, carbonic anhydrase, methanogens, cellulosome, nitrogenase, electrosynthesis, cutinase

## Abstract

Biotechnology has a high potential to substantially contribute to a low-carbon society. Several green processes are already well established, utilizing the unique capacity of living cells or their instruments. Beyond that, the authors believe that there are new biotechnological procedures in the pipeline which have the momentum to add to this ongoing change in our economy. Eight promising biotechnology tools were selected by the authors as potentially impactful game changers: (i) the Wood–Ljungdahl pathway, (ii) carbonic anhydrase, (iii) cutinase, (iv) methanogens, (v) electro-microbiology, (vi) hydrogenase, (vii) cellulosome and, (viii) nitrogenase. Some of them are fairly new and are explored predominantly in science labs. Others have been around for decades, however, with new scientific groundwork that may rigorously expand their roles. In the current paper, the authors summarize the latest state of research on these eight selected tools and the status of their practical implementation. We bring forward our arguments on why we consider these processes real game changers.

## 1. Introduction

**Figure 1:**
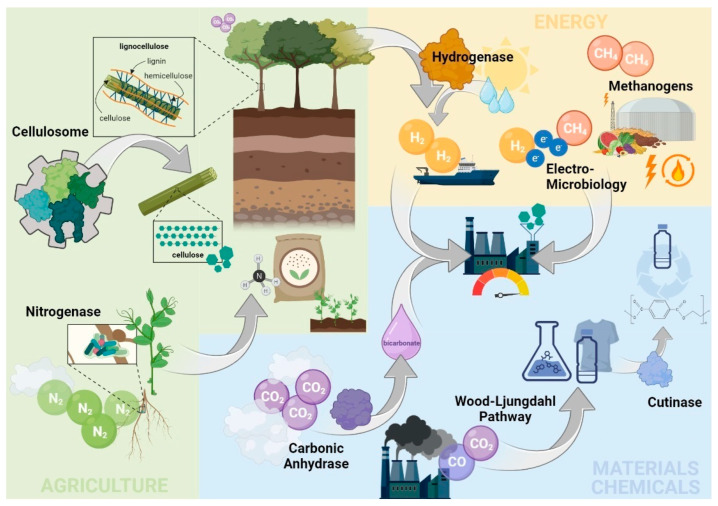
Overview on the eight up-coming biotechnological tools’ role in building a low-carbon bioeconomy within the agricultural (cellulosome and nitrogenase), energy (methanogens, electro-microbiology, and hydrogenase), materials, and chemical sector (Wood–Ljungdahl pathway, carbonic anhydrase, and cutinase).

Without a doubt, the climate crisis is one of the biggest threats faced by mankind today [1]. Ignorant of this fact, human activity produces greenhouse gas emissions (GHGs) at a record high, with few signs of slowing down. Global warming has a world-wide impact and will cause drastic changes in everyday life, depending on the actions taken. The challenges to substantially deliver on the targets agreed upon e.g., in the European Climate Law, will require fundamental transformations within all aspects of society, starting with how we grow food, use land, produce and transport goods, and power our economies [2]. While technological progress is one of the major contributors to climate change, new and efficient technologies can help us reduce net emissions and create a circular economy. Scalable new technologies and nature-based solutions are poised to propel us to a cleaner, more resilient world. High expectations are set on biotechnology as a sustainable alternative to the currently established industrial processes that often rely on fossil resources. With the exploitation of natural or genetically modified organisms and their metabolic capabilities, a wider range of industrial applications opens up. Nature’s blueprint for circular processes allows us to overcome the petrol-paved one-way street. Biotechnology is already well established amongst multiple bioeconomy sectors, including the agricultural, energy, materials, and chemical sector. Since the early days of discovery of a life not visible to the naked eye, biotechnological aspects have been in the focus of microbiologists. Luis Pasteur, a pioneer known for his achievements in identifying bacteria as the cause of diseases, devoted much of his work to understanding the nature of alcoholic fermentation. Beer brewing is the most widely recognized example of biotechnology’s traditionally strong role in food processing, while it also contributes significantly to the pharma and the chemical industry (lactic acid, citric acid or enzymes for e.g., laundry detergents). It is noteworthy that biotechnology is already a major player in environmental applications, as biological wastewater treatment is the state of the art, with an estimated 18,000 wastewater treatment plants (WWTPs) in operation in Europe and more than 14,000 in the U.S. [3,4]. In addition to millions of micro digesters, roughly 132,000 biogas plants of various sizes operate worldwide [5]. Bioethanol is another successful biotech example, with a global bioethanol production amounting to over 100 billion liters [6]. Brazil, being the pioneer for this renewable fuel platform, produces 30 billion liters of bioethanol annually, and thus covers 25% of its national fuel demand [7]. These examples demonstrate the importance of the biotech industry in continuing a green and sustainable development. There are numerous studies that underline the prominent role foreseen for biotechnology, especially when embedded in a biorefinery concept to serve a future low-carbon society. The authors fully support this point of view. In this paper we report on eight up-coming biotechnological tools that we think hold exceptional potential to mitigate the climate crisis within a low-carbon society (Figure 1). These tools are: (i) the Wood–Ljungdahl pathway, (ii) carbonic anhydrase, (iii) cutinase, (iv) methanogenesis, (v) electro-microbiology, (vi) hydrogenase, (vii) cellulosome, and (viii) nitrogenase. Some of them are fairly new and are explored predominantly in science labs. Others have been around for decades, however, with new scientific groundwork that may rigorously expand their roles. The selection is based on the potential of these technologies, when combined, to mitigate substantial amounts of GHG emissions within all major sectors of society on the way toward a low- to zero-carbon bioeconomy. Their substantial contribution to mitigate GHG emissions and future impact is estimated for each tool based on the available examples of large-scale applications within their respective sectors, presented in a separate chapter.

## 2. Selected Biotech Tools to Mitigate GHG Emissions with Major Bioeconomy Sectors

Biotechnology is already incorporated in many aspects of our everyday life and has been agreed to play a substantial part in future bioeconomy. We present eight up-coming tools with unprecedented potential to alter major industrial sectors that still rely on petroleum-based resources. The agricultural, energy, and materials and chemical industries are amongst the top GHG emitters, while also building the pillars of our economy. Thus, the selection comprises eight tools specifically targeting these industries in order to build a future low-carbon bioeconomy, independent of fossil resources.

### 2.1. Materials and Chemical Sector

#### 2.1.1. The Wood–Ljungdahl Pathway High-Throughput Microbial CO_2_ Fixation

One of the critical tasks within a circular economy is sustainably producing chemicals and fuels, whilst reducing GHG emissions. C1 biotechnology (bioconversion employing compounds containing a single-carbon atom) that utilizes GHG emissions as the sole carbon source enables carbon-negative manufacturing, the key process to achieve a circular economy [8,9]. Such C1 input streams include industrial flue gas from the steel and cement industries, syngas containing CO_2_, CO, and H_2_ from gasification of organic wastes and residual biomass, or CO_2_ from direct air capture. In nature, six metabolic pathways are known to fix inorganic CO_2_, the most prominent being the reductive pentose phosphate (Calvin–Benson) cycle used by cyanobacteria and plants. Alternative mechanisms for autotrophic growth exist in prokaryotes, all pivoting on acetyl-coenzyme A as a key metabolite [10,11]. Among these pathways, the reductive acetyl-CoA or Wood–Ljungdahl pathway (WLP), utilizing CO_2_ and H_2_, is thought to be the most ancient metabolic route. It is the only linear pathway and involves two metabolic branches to reduce two molecules of CO_2_ in parallel to form acetyl-CoA. It is also the only one that combines energy conservation with autotrophic biomass formation [12,13]. ATP is generated through a chemiosmotic mechanism and requires complex flavin-based electron bifurcation to transfer electrons [14]. Because of extremely low ATP yields, the WLP acts as a highly effective CO_2_ capturing system to compensate for the low energy generation per mole carbon. Its overall scheme is conserved in certain bacteria and archaea (e.g., methanogens, see also Section 2.2.1). However, only the carbonyl branch shares homology, whereas the archaeal and bacterial methyl branches involve different cofactors, electron transporters, and enzymes, making this system abundantly available for anaerobic organisms of all kinds [11]. Acetogenic bacteria, shortly termed acetogens, are phylogenetically diverse, belonging to 23 different genera [15]. They play a major role in the global carbon and H_2_ cycle, as fermentation processes have been developed to exploit their unique features based on gas streams of varying composition. Such biobased gas fermentation aims at the production of native bioproducts and chemical precursors such as lactic, and acetic acid, 2,3-butanediol, and ethanol [16]. Due to thermodynamic constraints, the native product spectrum of acetogenic strains from C1 gas is limited to only a handful of compounds [15]. The reduction of CO_2_ with H_2,_ or alternatively with CO, is limited and currently only two products, ethanol and acetate, are feasible at high titers. However, *C. autoethanogenum*, *C. ljungdahlii*, and *C. ragsdalei* were reported to rely on a different mechanism to conserve energy when ethanol is formed in large amounts from CO_2_ and H_2_. For these Clostridia, an enzyme that activates acetate with reduced ferredoxin as an electron donor, and the aldehyde–ferredoxin oxidoreductase acts as the driving force for ATP synthesis to overcome the energetic barriers, so that the overall process yields 1.2 ATP·mol^−1^ ethanol [17,18]. Moreover, the co-utilization of CO_2_ with CO was shown to enhance metabolic flux toward ethanol through the WLP [19]. While low CO concentrations significantly improved the overall gas fermentation performance and process stability, more than 50% of the captured CO_2_ was converted to ethanol by *C. autoethanogenum*, independent of CO supplementation [19]. The highest acetate titers of 40–60 g·L^−1^ were previously reported for batch-stirred tank reactor systems and continuous trickle-bed reactors using CO_2_ and H_2_ as substrates [20,21,22]. Syngas fermentation based on a mix of CO, CO_2_, and H_2_ yielded significantly lower acetate concentrations of 1.0–7.7 g·L^−1^ [23,24]. Acetate, however, is not the final product. Instead, it serves as a precursor to subsequently be converted to liquid fuels and chemical compounds. The microbial chain elongation of acetate to medium chain fatty acids (MCFAs), such as n-caproate, allows for further processing into a substitute for conventional aviation fuels [25,26], and other biochemicals and -materials. Effective elongation routes to MCFAs were previously demonstrated based on acidified organic (food-) waste [27,28]. In contrast, bioelectrochemical systems directly use CO_2_ for the production of MCFAs and alcohols by electro-autotrophic bacteria *Clostridium ljungdahlii* [29]. A common denominator in the chain elongation process is thought to be *Clostridium kluyveri* [30]. This anaerobic strain uses a unique ethanol–acetate metabolism to produce C4–C6 compounds (butyrate, caproate) and H_2_ [31]. Until recently, only a few gene-editing tools were capable of altering these microbes’ metabolism; however, the latest developments in synthetic biology of acetogenic model strains include ClosTron-targeted mutagenesis, transposon mutagenesis, homologous recombination, and CRISPR-Cas genome editing [32,33]. This new toolbox allows for the broadening of the currently limited substrate spectrum and pushing the acetogens’ native metabolism toward industrially relevant products at a high productivity. Metabolic engineering of known gas-fermenting Clostridia (*C. autoethanogenum*, *C. ljungdahlii*, etc.) recently enabled the efficient production of acetone, isopropanol, polyhydroxyalcanoates (3-hydroxybutyrate), and ethanol based on industrial emissions and syngas [34,35]. To date, LanzaTech is the leading company which exploits metabolically tweaked acetogens to produce industrially relevant chemicals and fuel precursors via the WLP on a large scale. LanzaTech established its first two commercial-scale gas fermentation plants in China, with an annual production of more than 90,000 tonnes [35]. Alongside LanzaTech, other competitors such as Coskata, Inc., Synata BIO, INEOS Bio, and JUPENG BIO, have been developing gas fermentation technology to convert synthesis gas from low-cost feedstocks into high-value products [36,37]. While Coskata, Inc. announced to cease operations in 2015 its syngas technology was incorporated by Synata Bio. To date, Costkata, Inc. remains the biggest patent-holding company in the world regarding syngas fermentation [38]. These examples demonstrate how advanced synthetic biology provides an efficient genetic toolbox for ancient microbes to keep gas fermentation on track. Such gas fermentation processes will in turn reduce global carbon emissions and serve the need for low-carbon biochemicals and -materials.

#### 2.1.2. Carbonic Anhydrase: An Efficient Enzymatic Capture of CO_2_

CO_2_ is the primary greenhouse gas emitted during natural and human activities [39]. The excessive use of fossil resources and its attributed CO_2_ emissions are the major cause of the climate crisis [40]. Global net anthropogenic greenhouse emissions have increased by 54% from 1990 until 2019, with 59 ± 6.6 gigatonnes of CO_2_ equivalent (CO_2eq_) emitted in 2019 [41]. Based on these numbers, the development of technologies to capture CO_2_ is an important pillar to effectively mitigate climate change [42,43]. In contrast to the WLP described in Section 2.1.1, the absorption in the liquid phase allows to efficiently capture and concentrate dilute CO_2_ present in the air or exhaust gas. With little alternative, the absorption of CO_2_ in the liquid phase is the most advanced technology that is deployed also at an industrial scale [44,45]. This process absorbs CO_2_ mostly into amine solvents such as monoethanolamine (MEA) and diethanolamine (DEA) that are predominantly used [42,44,45]. This amine absorption captures between 80 and 90% of the CO_2_ from exhaust gasses, but has a high energy requirement for solvent regeneration [42,46,47]. When applied to coal fire plants, this conventional carbon capture consumes around 30–40% of the energy output [48]. Another major drawback of amines as solvents is the side production of toxic waste [49,50]. Research aims at more sustainable alternatives to capture CO_2_ in non-toxic aqueous solvents. Various enzymes that fix CO_2_ are the focus of research interest [50,51]. Out of six major routes for CO_2_ fixation in living cells, two classes of enzymes have been identified for CO_2_ capture applications. The first is a class of oxidoreductases, including dehydrogenases, which use cofactors for the conversion of CO_2_ into various products. For example, NAD(P)H/NAD(P)^+^ act as electron donors and promote CO_2_ conversion into formate or methanol [52,53]. When H_2_ serves as an electron donor, the hydrogen-dependent CO_2_ reductase produces formate and combines CO_2_ capture with hydrogen storage [54,55] (see Section 2.2.3). Secondly, a class of lyases comprises the most prominent and most investigated member for CO_2_ capture, the carbonic anhydrase (CA) [42,56,57]. While the organisms involved in the WLP are stricly anaerobes, CAs are ubiquitously found in nature, including bacteria, fungi, plants, and mammals, and fixate CO_2_ aerobically [58,59]. They are classified as carbonate hydro-lyases and form a superfamily of metalloenzymes that genetically evolved independently into at least eight families, i.e., the α-, β-, γ-, δ-, ζ- η-, θ- and ι-CAs [60]. CAs play crucial roles in numerous physiological processes such as pH homeostasis, respiration and the transport of CO_2_/bicarbonate (HCO_3_^-^), calcification, electrolyte secretion, and many more [61,62,63]. Moreover, CAs are responsible for concentrating CO_2_ near RuBisCo, the essential enzyme for CO_2_ fixation in plants. Highly efficient CAs that were introduced in microalgae significantly enhanced their biomass and lipid production, and thus pose a potential strategy for biofuel production from atmospheric CO_2_ [64]. CA speeds up the hydration of CO_2_ occurring naturally when CO_2_ is brought into aqueous solutions [65], and is one of nature’s fastest enzymes, with a turnover rate of 10^4^–10^7^ s^−1^ [66]. This accelerated reaction enables a fast and complete CO_2_ capture in non-toxic aqueous solutions. In contrast to the widely used chemical absorption currently employed at an industrial scale, simple salt solutions or even plain water can replace toxic amine solvents [49,67]. The heat of adsorption is an indicator for the overall energy demand of such absorption processes. Compared to 1.92 GJ·t^−1^ CO_2_ for MEA, this value is significantly lower for K_2_CO_3_ and water, at 0.64 and 0.39 GJ·t^−1^ CO_2_, respectively [68], resulting in a more economic process overall. More than 100 new patents related to CA-assisted carbon sequestration were registered between 2011 and 2022, showcasing the high relevance of the field [66,69,70,71,72,73]. Still, conditions are harsh during enzymatic CO_2_ capture applications. At the absorber the prevailing temperature is between 30 and 50 °C, while it is increased up to 80–100 °C in the stripper to release the CO_2_ gas [74]. The free-floating CA is recycled together with the solvent from the absorber to the stripper and back, thus exposing the enzymes to strongly varying temperatures. Therefore, an enhanced thermal stability of native and bioengineered CAs is crucial and in the focus of current research [67,75,76,77]. In an alternative strategy, CAs are immobilized during the absorber stage. The advantage is that the enzymes are not exposed to the high temperatures in the stripper. Because of that, enzymes can be reused more often at an increased long-term stability [78,79,80,81,82]. The feasibility and implementation of such an enzymatic process at the industrial scale was demonstrated by the Canadian company CO_2_ solutions Inc. at their pilot facility in 2017. Throughout more than 2500 operation hours, 10 tonnes of CO_2_ were captured per day and the CO_2_ product stream reached a purity of over 99.95% [74]. In 2020, the Italian company Saipem purchased the CO_2_ capture technology, including 90 patents from CO_2_ Solutions Inc. [83]. Another example of a successful scale-up of enzymatic CO_2_ capture was presented by the American company Akermin Inc., which worked on immobilized CA technology from 2010 to 2013. Akermin Inc. demonstrated 90% capture at ~19.5 Nm^3^·h^−1^, and ~80% capture at ~ 30 Nm^3^·h^−1^ for more than 2800 operation hours, with a minimal decline in enzyme activity [84].

#### 2.1.3. Cutinases: Old Enzymes with a High Relevance for the Future

The leaves and fruits of higher plants are protected by a polymeric cuticle, which is composed of the natural polyester cutin embedded in cuticular wax [85]. This layer is essential in keeping the aerial parts of the plant waterproof and safeguarding them against damage and stress. It is also the initial barrier that microbial plant pathogens must penetrate to utilize the plant’s carbon sources [86]. To invade plants, pathogens produce cutinases, which are enzymes capable of breaking down cutin through the hydrolysis of its polymeric network. Cutinases are primarily produced by plant pathogenic fungi, but also by some bacteria and plants [87]. As serine hydrolases, cutinases cleave ester bonds such as esterases and lipases. Most cutinases characterized to date are versatile and can process a range of substrates under neutral-to-alkaline conditions, and temperatures between 30 and 60 °C [88]. With a high stability and catalytic efficiency, cutinases have become popular industrial enzymes due to their ability to make industrial processes more environmentally friendly [87]. The success of cutinases can be traced back to the textile industry, where they are used for “bioscouring” cotton fibers [89]. These fibers are mostly composed of cellulose, but are coated with a hydrophobic cuticle that includes cutin, wax, pectin, and proteins. The scouring process removes non-cellulosic components that account for up to 10% of the total fiber weight, improving the fiber’s wettability and enabling uniform dyeing and finishing [90]. The traditional scouring process uses sodium hydroxide at 100 °C, consuming large amounts of water and energy and damaging the fibers. Bioscouring with cutinases, along with cellulases, pectinases, lipases, and detergents, is a more environmentally friendly alternative that can be performed at low temperatures, uses less energy, and saves up to 50% of the water used in chemical treatments [91,92,93,94]. Bioscouring can also be combined with other wet processing procedures such as bleaching and biopolishing in a one-step process to save 350 kg of CO_2_ and 13 m^3^ of water per ton of fabric [95]. Cutinases are also a vital ingredient in modern detergents to enhance stain removal [96]. The energy required to heat the water during washing has the highest environmental impact of detergents. Cutinases are active in aqueous solutions and water–oil emulsions, and are applied in detergents to remove fat-based stains. Thus, enzymes such as cutinases allow for washing at lower temperatures, and using biodegradable multienzyme blends in detergents can save up to 10 g of CO_2_ per wash and reduce the amount of chemicals in our aquatic environments [97]. The greatest potential for cutinases lies in boosting the circular use of polyesters, such as polyethylene terephthalate (PET), which is one of the most widely used synthetic plastics, with an annual production of 73.4 megatonnes in 2021 and a projected 104.1 megatonnes by 2028 [98]. Although mechanical PET recycling has a 41% average rate in the EU (corresponding to 14.1 kg per capita), it is limited by the polymer chain length reduction and contamination, leading to downcycling from food-grade to application as fleece fibers [99]. Monomer recycling allows for an infinite re-use of the monomeric building blocks and low-molecular-weight components. Cutinases have shown the ability to degrade not only cutin, but also synthetic aromatic polyesters such as PET [87]. This makes cutinases and related α/β hydrolases, such as esterases and lipases, crucial in green chemical recycling and functionalizing polyester surfaces to change superficial properties, such as hydrophobicity or hydrophilicity. Cutinases can hydrolyze PET at temperatures of 40 °C to 70 °C and pH 7 to 9, releasing oligomers and monomers such as terephthalic acid and ethylene glycol. Thermostable cutinases from different strains, such as Humicola insolens cutinase [100], Thermobifida fusca cutinases [101], and leaf–branch compost cutinase [102], have been isolated and their activity improved by modifying the active site, reducing inhibition from release products, and increasing stability [103,104]. The cleantech company, Carbios, founded in 2011, is planning the world’s first plant for biologically recycled PET in France. The demonstration plant in Clermont-Ferrand, set to begin operation in 2023, will depolymerize 60 tonnes of PET, equivalent to about 3.2 million plastic bottles [105].

### 2.2. Energy Sector

#### 2.2.1. Methanogens: Suppliers of Renewable Natural Gas

The most striking feature of methane, with respect to climate change, is its highly ambivalent character. On the one hand, it is considered an appealing renewable energy carrier, extracting the calorific value from waste materials. On the other hand, it is a potent greenhouse gas with an impact 28 times higher than CO_2_ [106]. It is estimated that methane has accounted for roughly 30% of global warming since pre-industrial times. Cutting methane emissions is considered the fastest opportunity to immediately slow the rate of global warming [107]. More than 70% of all methane emissions are of biological origin, including the significant anthropogenic sources of livestock breeding and agricultural practices [108]. The first indication that methane gas can be biologically produced was credited to Alesandro Volta in 1776, who hypothesized that flammable swamp gas is derived from decaying organic matter [109]. Meanwhile, this natural anaerobic digestion process eventually leading to the formation of biogas is well understood. It involves a series of steps performed by microbial specialists. The final conversion step—the formation of methane—is the contribution of a unique group of microorganisms termed archaea. The story of archaea is unique. It was only in the late 1970s that Carl Woese established this unique group of microorganisms classified as one of the three domains in the Tree of Life, based on 16s RNA analyses. Archaea are renowned to comprise several specialists which can survive under extreme conditions. Despite their presence in exceptionally harsh environments, they are ubiquitous in nature [110]. They possess a unique set of structural and functional molecules that enable them to exist at a high temperature, pressure, and salinity. The features that distinguish them from bacteria include unique composition of lipids, cell wall structures, and eukaryotic-type DNA-dependent RNA polymerase and DNA replication systems. Several homologies with eukaryotic genes suggest that an archaean cell stood at the beginning of the eukaryotic lineage [111]. Archaea are among organisms that exhibit a high biotechnological potential for various applications [112,113,114]. Methanogenesis as a mechanism for energy conservation is exclusively attributed to the archaeal domain. There are five major pathways of methanogenesis [115], among which are methylotrophic, methoxydotrophic, and alkylotrophic. These pathways all share the key enzyme methyl-coenzyme M reductase (MCR), which catalyzes the final formation of methane [116]. As a side note, this enzyme also plays an important role in the reverse reaction called the the anaerobic methane oxidation (AMO). The AMO-associated methanotrophs are also members of archaea and play an important role in the mitigation of climate change by reducing methane emissions from anaerobic environments [117]. AMO is a methane-consuming microbial process occurring in anoxic marine and freshwater sediments, where sulfate, nitrate, nitrite, or metals, such as manganese, serve as alternative terminal electron acceptors [118,119]. Huge research effort has been put into studying the role and difficile interaction of methanogens with other microbial groups. In such a syntrophic interaction between fermentative bacteria (syntrophs) and methanogenic archaea (methanogens) the immediate transfer of H_2_—or redox equivalents in general—between the two partners opens a thermodynamic window which allows both species to conserve energy from the overall reaction [120]. Biogas formation is a traditional and widely established way of climate-friendly energy production through the anaerobic digestion of biomass. Increasing knowledge has improved process stability, reduced operational failures, and broadened the substrate range for biogas production. Elucidation of the catalytic mechanism of the involved key enzymes and the development of metabolic engineering tools are setting the stage for advanced applications of anaerobic digestion [121]. Today, there is around 18 GW of installed power generation capacity running on biogas around the world, and the capacity increased on average by 4% within the last decade [122]. A recent report of the world biogas association claims that anaerobic digestion replacing conventional fossil energy carriers has the potential to reduce global GHG emissions by 3290 to 4360 Mt CO_2eq_, which is equivalent to 10–13% of the world’s current GHG emissions [5]. Besides this well-established technical utilization of methanogens, new applications have come into focus. One such approach is biological biogas upgrading to biomethane. In 2018, almost two-thirds of biogas production were used for on-spot electricity and heat generation [122]. However, the future lies in the injection into the gas grid serving as storage for intermittent energy. Biomethane serves as a perfect substitute for natural gas and allows for the utilization and easy distribution through the existing infra-structure of the natural gas grid. To meet quality criteria—in particular the calorific value of natural gas—the methane concentration has to be increased to at least 80%, depending on the country-specific legal requirements. Various physico-chemical technologies for such biogas upgrading through CO_2_ removal are available. An alternative approach is applied in biogas upgrading, where electrolytically produced H_2_ is utilized to microbiologically convert the CO_2_ fraction in biogas to CH_4_, yielding >95% of overall carbon conversion to methane [123]. A direct injection of H_2_ into an anaerobic digester, in situ methanation, is one option for this upgrading technique. The other attempt is the post-treatment of the biogas in an external bioreactor, referred to as ex situ methanation [124]. In place of biogas upgrading, the methanation of CO_2_-rich gas may also be employed. Despite certain transfer losses, this power of the gas concept is considered the most cost-efficient long-term storage option for intermittently produced electric power [125]. A promising approach is the utilization of hyperthermophile pure cultures at hyperbaric pressures, which has been demonstrated to exhibit high volumetric productivity [126,127]. Several demonstration projects for the described technologies are on their way. According to a recent survey, in the foreseeable future, 19 MW of installed electrolyzer capacity will be connected to biological methanation [128]. In contrast to catalytic methanation, the biological process is more robust toward impurities and intermittent substrate gas supply, but demands a bigger reactor dimension due to lower volumetric yields [129]. In geo-methanation concepts, depleted gas reservoirs serve as natural underground bioreactors, allowing for overcoming the limitation of scale [130]. A study reports two demonstration projects which investigate the feasibility of this approach: HyChico in Argentina and Underground Sun Conversion (USC) in Austria [131]. An even more futuristic approach considered cutting-edge in research is the utilization of direct electron transfer to methanogens, a concept termed electro-methanogenesis (see Section 2.2.2) [132].

#### 2.2.2. Electro Microbiology: Merging Electricity and Biology

The connection between electricity and life was first discovered in the early days of modern electricity. Luigi Galvani’s famous experiment with dancing frog legs was a result of saltatory conduction, involving the propagation of electric action potentials along the nerves. Today, microbiologists are interested in a modified version of ‘animal electricity’ known as electroactive microbes. These microbes have the ability to shuttle electrons across their cell membranes and extracellularly transfer them to or from an electrode in the surrounding liquid [133]. Such redox reactions drive almost all metabolic activity in a cell. This ability helps them harvest energy through reducing or oxidizing insoluble electron acceptors or donors in their native environment. First reports on such observations date back more than a century [134]. Since then, several practical applications with a high potential in the biotech industry have attracted scientific interest [135]. The mechanism behind this process involves different ways of transferring electrons across a cell membrane, including indirect electron transfer via soluble electron carriers, or direct electron transfer through membrane-bound proteins or conductive nanowires (pili). The latter act faster and are primarily associated with different species of Geobacter [136]. Multiheme c-type cytochromes are now identified as the key players in electron transfer [137], playing a crucial role in cellular respiration and the Great Oxidation Event 2.3 billion years ago, when the Earth’s atmosphere changed drastically [138]. Cytochromes are redox-active proteins that operate within an impressively wide range of electrochemical potentials, with heme cofactors in their active centers responsible for the efficient electron transfer. Electro-active bacteria have a huge potential for exploitation [139,140], particularly in the field of renewable energy production, through microbial fuel cells [141], electro-fermentation, and electro-methanogenesis. Microbial fuel cells harvest electric energy through chemical reactions, similar to their non-biogenic counterparts. The microorganisms themselves catalyze the process and transportation of electrons, making the requirement for a chemical catalyst obsolete. Although the improvement in the electrical performance of microbial fuel cells was remarkable in the last years, it is still insufficient for implementation at an industrial level [142]. In electro-fermentation, an electric current is used to control the microbial metabolism and drive otherwise unfavorable reactions through a change in the redox balance using external electrodes. These electrodes serve as either electron sinks or sources, thus allowing for an unbalanced fermentation to modify the product spectrum. In microbial electrolysis, for example, electrochemically active bacteria can convert organic matter into hydrogen when electric energy is added to the system to overcome thermodynamic barriers [143]. Supplying a small voltage may gain a theoretical maximum of 12 mol H_2_ per mole of glucose, whereas conventional oxidation would result in only four moles of hydrogen [144]. The combination of wind turbines or solar panels with wastewater treatment is also being explored as a way to generate H_2_. This synergistic process requires less energy input than water electrolysis, as it exploits the biochemical energy of organic waste. Electro-methanogenesis, already mentioned in Section 2.2.1, is another promising application wherein electrons directly enable CO_2_ conversion through methanogens [145]. Although technical challenges still need to be overcome, companies such as Electrochaea or Cambrian Innovation [146] are currently developing large-scale reactors [132]. Electrotrophic microorganisms present at the cathode catalyze the conversion of CO_2_ into alcohols, acids, and other organic molecules, and provide reducing power for the valorization of syngas or CO_2_ streams. As demonstrated, microbial life can be fueled solely by electricity [147]. The integration of electronics and biology can be greatly enhanced through the use of electroactive microorganisms, thanks to recent advancements in system, cell, and molecular biology. Another illustration of this integration can be seen in biological sensors, which have the potential to offer advanced analytical tools across various fields, including environmental monitoring [148]. The application of biosensors in climate-smart agriculture is crucial for addressing the challenges posed by climate change and promoting both agricultural and environmental sustainability [149].

#### 2.2.3. Hydrogenases: A Key Enzyme behind the Green Hydrogen Economy

Hydrogenase is an enzyme that catalyzes one of the simplest chemical reactions, the reversible interconversion of hydrogen to protons and electrons [150]. Hydrogenases likely evolved during the earliest life to exploit the hydrogen-rich atmosphere of the young Earth for energy [151]. Today, hydrogen is a crucial component in the transition to a clean and non-fossil energy future, particularly in hard-to-decarbonize sectors such as heavy industry, steel manufacturing, chemical production, heavy-duty road transport, shipping, and aviation. According to a recent survey by the International Energy Agency (IEA), 17 governments have released hydrogen strategies, while more than 20 governments have announced their intention to develop such strategies, and many companies are seeking to exploit opportunities in the hydrogen business [152]. Currently, many technological solutions, such as electrolyzers, membrane fuel cells, and photoelectrochemical cells, rely on the powerful catalytic properties of platinum metal. However, hydrogenases have been shown to rival platinum, working at a thermodynamic equilibrium and with high catalytic rates. Their use as substitutes for noble metal catalysts in future technological devices holds promise for the development of a sustainable and economically viable H_2_ economy [153]. Hydrogenases play a vital metabolic role in many different aerobic and anaerobic microorganisms. They remove excess reducing power generated during metabolism by evolving hydrogen, or can oxidize hydrogen to generate reducing power for growth [154]. These enzymes are widespread in bacteria and archaea and are even found in some Eukarya [155]. The families of Thermococcaceae and Clostridia are renowned for their high H_2_ uptake [124] or H_2_ production rates [156,157], respectively. Hydrogenases are classified into three phylogenetically distinct groups, depending on the type of catalytically active metal center: [NiFe, [FeFe], and [Fe] hydrogenases [158]. [NiFe] hydrogenases are of the hydrogen uptake type, [FeFe] hydrogenases mainly produce H_2_, and [Fe] hydrogenases catalyze specific reactions utilizing H_2_. Hydrogenases may also form part of quaternary enzyme structures and multienzyme complexes. Examples include the energy-converting hydrogenases (Ech) of methanogenic archaea [159] and acetogenic bacteria [160], which link microbial energy metabolism to H_2_ metabolism [161]. Today, many microorganisms possess complex respiratory chains composed of multiple different components. However, several archaeal or bacterial species use a simple respiration process comprising only a hydrogenase and an ATP synthase for energy conservation [160]. The numerous biotechnological opportunities regarding microbial H_2_ utilization for CO_2_ reduction to CH_4_ or acetate are widely recognized [126,127,159] and are discussed in Section 2.1.1 and Section 2.2.1. Hydrogenases are also involved in other multienzyme complexes such as flavin-based electron bifurcation, a recently discovered mechanism of biological energy coupling [162]. Moreover, H_2_ generation is closely linked to N_2_ fixation, where a hydrogenase recycles the produced H_2_ to minimize the loss of energy during nitrogenase catalysis [163]. Hydrogenases also play a crucial role in the production of hydrogen during nitrogenase catalysis [163] and biological formation of H_2_. There are two main ways to generate biohydrogen from renewable resources: through the “dark fermentation” of low-cost substrates and waste, or “biophotolysis” [164]. In the latter, hydrogen is produced through the photosynthetic splitting of water by green algae or cyanobacteria. This reaction only requires water and light, and it produces no greenhouse gasses, making it the ultimate method for hydrogen production [165]. Researchers have been working to increase the efficiency of biohydrogen formation, for example, through genetic engineering. However, while biohydrogen production has shown promising results at the pilot and laboratory scales, studies on scale-up to the industrial level are still lacking [166]. Hydrogenases can also act in cell-free systems, serving as excellent electrocatalysts when attached to electrodes. For example, a system composed of [FeFe] hydrogenase embedded in a modified hydrogel has been used to achieve reversible hydrogen conversion, with a voltage of 1.16 V near the thermodynamic limit [167]. There is ongoing research into the commercial exploitation of these approaches in a variety of bio-enhanced systems, such as fuel cells, hydrogen production devices, and electrocatalysis [153,168,169]. Another hydrogenase of biotechnological interest is the ‘hydrogen-dependent CO_2_ reductase’ (HDCR), which catalyzes the oxidation of hydrogen coupled to the reduction of CO_2_ to form formate (see Section 2.1.2). HDCR is a filamentous enzyme that self-assembles in a linear manner, enhancing electron transfer and its activity. This supramolecular organization forms a specialized metabolic subcompartment within the cell [170]. HDCR operates at ambient temperatures and pressures, and reduces CO_2_ more effectively than any other known biological or chemical catalyst. Additionally, it is tolerant against carbon monoxide, a frequent contaminant in flue gas, making it a topic of interest in two fields relevant to climate change: reversible hydrogen storage and CO_2_ capturing [55].

### 2.3. Agricultural Sector

#### 2.3.1. Cellulosomes: Extracellular Nanomachines for Dismantling Plant Polysaccharides

The conversion of lignocellulosic biomass into chemicals has attracted the attention of numerous researchers with the aim of establishing a sustainable economy independent of fossil resources. Lignocellulose as a feedstock provides several advantages over other biomass: it is abundant, renewable, and, not to forget, inedible, at least for humans. The latter being an essential asset to circumvent the so-called food or fuel debate [171]. It is estimated that the amount of lignocellulose produced by the photosynthesis of plants on earth is approximately 2 × 10^11^ tonnes per year [172]. Easily accessible C6 and C5 sugars build the backbone of cellulose and hemicellulose—two of the three main components of lignocellulose—respectively. This polysaccharide structure makes them highly suitable substrates for biorefinery processes. However, prior to consumption by microorganisms, the lignocellulose must be broken down into water-soluble fragments by exoenzymes. Microbial textbooks generally describe exoenzymes as active proteinaceous substances that are secreted by microorganisms into their local environment. For reasons of substrate competition, the generated products should remain as close as possible to the cell surface for immediate uptake. A sort of ‘leash’ that retains the enzymes in the cell vicinity would therefore make perfect sense. An exact such a structure was found for cellulose-degrading enzymes in *Clostridium thermocellum*. Such cellulosomes are self-assembled multienzyme complexes that are highly efficient at degrading cellulosic compounds. This cell-bound complex orchestrates the deconstruction of cellulose and hemicellulose [173]. The discovery of the cellulosome started with the observation of *C. thermocellum* attaching strongly to the insoluble cellulose substrate prior to its degradation. In the search for the cellulose binding factor instead of a small protein, a large multi-subunit supramolecular complex was isolated [174]. The functional catalytic compounds in such a supramolecular complex are attached to non-catalytic structural scaffolding proteins. The assembly between the backbone and the enzyme occurs via the interaction of so-called cohesins that are located on the scaffold and dock to the modular components of the enzymes. Two kinds of cohesin–dockerin complexes are responsible for enzyme integration into the cellulosome and tethering to the bacterial wall, respectively [175]. The cellulosome is one of the most efficient machineries for the degradation of plant cell walls, and it can potentially be used for the large-scale conversion of biomass [176]. Today, cellulosomes have been confirmed in several, but not all cellulolytic bacteria. There has been a rapid expansion in the identification of cellulosomes from various cellulolytic strains, revealing a surprising diversity in the composition and architecture of the component parts. Moreover, cellulosomes exhibit a distinct variability in their substrate spectrum. They degrade not only crystalline cellulose, but also hemicelluloses, chitin, and even pectin, depending on the source of the cellulosome [177]. It is no wonder that exploitation of the many features of cellulosomes has attracted the attention of several scientists. It has been suggested to take cellulosomes apart and reassemble their components to design artificial complexes [178]. One such attempt addressed the enhancement of alkaline stability. Utilizing the cellulosomal systems of *C. alkalicellulosi* would facilitate the breakdown of lignocellulosic material following alkaline pretreatment. Another attempt enhanced the thermostability, which thus allows for usage at higher temperature levels [179]. Meanwhile, the mechanistic understanding and the ability to manipulate cellulosomes has come quite far. A review paper from 2015 lists eleven different published approaches to construct synthetic cellulosomes [180]. Techniques that utilize whole-cell conversion are particularly promising to fully exploit the potential of lignocellulosic biomass. Therein, anaerobic cellulolytic bacteria are grown directly on the substrate instead of using an enzyme extract [181]. When taking a look at commercial opportunities, cellulases have a well-established market, at USD 1500 million in 2019. It is expected to reach USD 2320 million worldwide by 2024, showing a proportional increase across varied sectors, including animal feed, textile industry, food and beverages, and biofuels [182]. So far, cellulosomes do not play a significant role in this business. Currently available commercial cellulases are conventional (soluble) exoenzymes predominantly produced by fungal hosts belonging to *Trichoderma* spp. and *Aspergillus* spp. [183]. Nevertheless, the unique features of cellulosomes have tremendous potential for cost reduction and are considered a potential game-changer in the biofuel production and lignocellulose-based biorefineries. It has also been demonstrated that a cocktail combining free cellulolytic enzymes and cellulosomes displays a dramatically enhanced synergistic activity. While the free enzymes are more active on pretreated biomass, cellulosomes physically separate individual cellulose microfibrils from larger particles, resulting in an enhanced access to cellulose surfaces [184].

#### 2.3.2. Nitrogenase: A Bio-Based Approach to Substitute the Energy-Intensive Haber–Bosch Process

By 1913, Haber and Bosch succeeded in converting atmospheric nitrogen (N_2_) to ammonia by forcing it to react with H_2_ in a high pressure, high temperature reactor, and establishing the process at an industrial scale. About half of the global food production is tied to the usage of commercial fertilizers which rely on this process [185]. Today, the Haber–Bosch process produces about 135 megatonnes of ammonia every year, corresponding to over 400 megatonnes of CO_2_, i.e., about 1.2% of global CO_2_ emissions [186]. It is given that in a world where agricultural products and lignocellulosic material shall replace fossil resources, nitrogen demand will increase even further [187]. Some billion years ago, a smarter version of the Haber–Bosch process was invented by prokaryotes, taking place at an ambient pressure and temperature. These organisms developed enzyme-based nitrogen fixation in an environment running short of nitrogen compounds to tap the vast reservoir in the atmosphere. Nitrogenase is a metalloenzyme complex composed of the catalytic component, dinitrogenase, and an ATP-dependent electron-donating protein, the dinitrogenase reductase. The catalytic domain of the most widely distributed class of di-nitrogenases contains a molybdenum–iron cofactor, while other forms contain vanadium or iron only, without hetero-metal atoms. The three different cofactors in the active site of the nitrogenase complex are among the most complex metal-based cofactors in nature [188]. Nitrogenase catalyzes half, if not more, of nitrogen fixation on earth today [189]. A diverse group of free-living, plant-associative and symbiotic prokaryotes are able to perform biological nitrogen fixation through this mechanism. Noteworthy, the taxonomic distribution of nitrogenase is restricted to bacteria and archaea, with no known examples of the nitrogenase-encoding genes occurring within the Eukarya domain [190]. Within archaea, nitrogenase is restricted to only a few species. From the agricultural point of view, the most important process is symbiotic nitrogen fixation, which involves the endosymbiotic *Rhizobium* species residing inside the nodules formed on the roots of the host plant. The bacteria reduce atmospheric nitrogen, supplying ammonia to the host in exchange for carbon sources and energy. The plant partners of Rhizobia belong to the *Leguminosae* family. Soybean represents the most important and cultivated legume, the acreage of which reached 131 million ha in 2017 [191]. Rhizobia have been studied for over 100 years because of their ability to increase yields of legume crops. The first industrial production of rhizobial inoculants began by the end of the 19th century. Since then, farmers have applied such biofertility inoculants in organic crop systems, and as an alternative to expensive chemical fertilizers, and the business has turned into a billion-dollar market [192]. However, several important crop plants are non-leguminous and are unable to form symbiotic associations. Three biotechnological approaches have been envisioned to increase biological nitrogen fixation for non-legume species, in particular for the most popular cereal crops such as rice, wheat, and maize [193]. In the first strategy, bacteria that are already naturally associated with plants are modified to improve their colonization ability, overall growth, and N_2_-fixing capabilities. The second strategy aims to engineer non-legume cereal crops to form nodules, providing an adequate environment for N-fixing bacteria. The third strategy involves the transfer of prokaryotic nitrogenase genes into the plant genome itself. Such an advanced approach has been adopted recently. Rather than targeting the nuclear DNA, the organelles within the cell—specifically, the chloroplasts and the mitochondria—are genetically modified. It seems much easier to transfer genes from a prokaryote to a prokaryote-like system than re-engineer a whole pathway into a eukaryote [194]. One study describes the development and deployment of the first microbial product (*Klebsiella variicola*) optimized using synthetic biology to enable enhanced biological nitrogen fixation for corn [195]. The successful expression of the minimal N-fixing gene cluster from *Paenibacillus* in a model plant represents another major breakthrough in N-fixation research [196]. To date, despite significant progress, none of the attempts have fully succeeded. Scientists are confident that combining the prospecting of plant and bacterial natural diversity with genetic engineering will deliver solutions to replace energy intensive artificial fertilizers in the mid and long term [197]. In similarity to legume symbioses, there are actinorhizal plants forming N_2_-fixing root nodules in association with the *Frankia* bacteria [198]. Most actinorhizal plants are woody shrubs or trees. Symbiotic di-nitrogen fixation by trees can either economically contribute to the extended biomass requirement to substitute fossil resources or directly fix carbon emissions as woody biomass [199,200]. A N-fixing, also termed diazotrophic, microorganism may also be employed in gas fermentation (see Section 2.1.1), where N_2_ as an essential nutrient could be supplied via the gas phase. Thus, the two most essential elements for microbial growth—C and N—may be delivered through gaseous feedstocks [201]. This might be of particular advantage when extending the product range to nitrogen compounds such as amines or anilines. Nitrogenase has even more to offer. In fact, nitrogenases have one of the broadest substrate spectra known for enzymes. Besides their natural N_2_ substrate, they can reduce a wide range of small carbon/nitrogen compounds [202]. The conversion of CO_2_ and CO into methane and higher hydrocarbons in particular, suggests an industrial use of nitrogenases for carbon capture and the production of biofuels within a green economy. Consequently, nitrogenases have the potential to replace two important industrial processes at once: the Haber–Bosch process for fertilizer production, and the Fischer–Tropsch process for fuel production [203]. For the latter, most of the development is at a fundamental stage and its practical application is by far not as advanced as acetogenic syngas fermentation. Nevertheless, scientists are advancing even further. A nitrogenase variant that converts carbon dioxide to methane has been expressed in an engineered strain of the photosynthetic bacterium *Rhodopseudomonas*, opening a door to the light-driven conversion of CO_2_ to value-added commodity chemicals [204].

## 3. Potential Impact of Selected Biotechnological Tools

How big of an impact can the technologies and applications described actually have? The following paragraph compiles facts and figures that underline the huge global potential of the described microbial tools for greenhouse gas (GHG) mitigation. The given numbers are based on available examples of large-scale applications within their respective sectors.

### 3.1. Wood–Ljungdahl Pathway

Syngas fermentation utilizing acetogenic bacteria provides alternatives for green chemicals aiming to substitute their petrol-based counterparts. The carbon (C) demand for chemicals and derived materials is estimated to be 450 megatonnes [205]. Alongside point-source CO_2_ emissions (steelworks, power stations, cement and lime production, etc.), the most reasonable and available input materials for gasification systems to produce this syngas formation are municipal solid waste and sewage sludge. Lignocellulosic material has also been listed as potential feedstock, but the recovery of its sugar content within a lignocellulosic biorefinery seems more straightforward. On a global level, municipal solid waste amounts to roughly 1.3 megatonnes per year. With an average C content of 270 kg·t^−1^, this corresponds to 300 megatonnes C [206]. The global production of sewage sludge from municipal WWTPs is 45 megatonnes dry mass per year [207]. Calculating with a C content of 390 kg·t^−1^ [208], this corresponds to 17.5 megatonnes C. Accordingly, these two sources are sufficient to cover almost all C required for green chemical production. Additional substrate sources for gas fermentation are point-source streams such as refinery off-gas and steel or ferroalloy off-gas, with a yearly potential of around 13 and 78 megatonnes C, respectively [209]. Ethanol, through syngas fermentation, is the first industrial application that is already established at the large scale, with butanol and acetone being the next two candidates for which pilot trials are on their way [35]. The current market size of these three chemicals is 80, 5, and 7 megatonnes, respectively, equivalent to a total of 49.3 megatonnes C (or 181 megatonnes CO_2_ captured). The raw materials for these chemicals are currently petrol-based or purpose grown crops. In the latter case, a switch to MSW or sewage sludge would save these natural resources for feed/food or other high-level purposes.

### 3.2. Carbonic Anhydrase

Carbon capture and the subsequent storage or utilization (CCUS) plays a prominent role in all scenarios reaching net-zero emissions. The IEA designed a ‘Sustainable Development Scenario’ with technology needs and opportunities for reaching international climate and sustainable energy goals [210]. According to this scenario, 840 megatonnes CO_2_ shall be captured in 2030, of which 189 megatonnes will be used; in 2070, when the net-zero emissions are reached, the respective figures should be 10.4 gigatonnes CO_2_ captured, of which 0.9 Gt will be used. CCUS deployment has been behind expectations in the past, but momentum has grown substantially in the recent years. There are around 35 commercial facilities applying CCUS to industrial processes, fuel transformation and power generation, with a total annual capture capacity of almost 45 megatonnes CO_2_ [210]. The types of technology vary by industrial sector, with post-combustion with amine adsorption and chemical loop technology being the most commonly cited [211]. With consideration of the current state of development, it is not possible to predict to which extent modified adsorption technologies employing carbonic anhydrase will play a role amongst the many competing technologies. However, given the predicted important contribution of CCSU to a zero-emission society, the overall potential is considered to be particularly high [212].

### 3.3. Cutinase

Among the proposed tools, cutinases probably have the smallest, nevertheless still considerable, GHG mitigation potential. PET is the most abundant polyester manufactured globally. About 56 megatonnes of PET are produced annually and it is typically recycled at a rate of nearly 25% [213]. Although this is the highest recycling rate of all plastic categories, product quality and supply chain economics constitute certain limitations. Tertiary recycling, i.e., decomposing waste plastic into its building blocks which subsequently serve as feedstock for the production of new plastic material, allows for the application of improved refining methods to reestablish the original quality [214]. So far, chemical recycling is relatively inefficient, but the utilization of cutinases may significantly enhance the economics of the process. According to a review study, the emission factor of PET, i.e., saving of kg CO_2_ per ton recycled material, is around 1570 [215]. Assuming an increase in the recycling rate by 10 percentage points (from 25 to 35%) and applying a diminished emission factor of 1000 kg·t^−1^ (taking into account the increased effort for tertiary recycling), the savings would be 5.6 megatonnes CO_2eq_.

### 3.4. Methanogens

The best estimates of the potential impact can be made for the case of methanogens. The biogas and biomethane industries are significant and growing contributors to achieve climate neutrality by 2050. A potential of 38 bio m^3^ biomethane is estimated from anaerobic digestion by 2030 for EU-27, increasing to 91 bio m^3^ by 2050 [216]. This is a substantial contribution, as the 2020 EU natural gas consumption was 412 bio m^3^ in 2021 [217]. Another report estimates that by 2040, over 200 bio m^3^ of biomethane could be produced worldwide. Still, most biomethane production today takes place in Europe and North America, with Asian countries catching up rapidly. The full utilization of the sustainable potential could cover 20% of today’s worldwide gas demand [122]. According to a white paper, the sector has the potential to reduce worldwide emissions by 3290 to 4360 megatonnes CO_2eq_, which is equivalent to 10–13% of total GHG emissions [5]. Moreover, power-to-methane converting excess renewable energy is an up-coming technology with a high potential. It is one of the few viable options for large-scale energy storage using existing gas infrastructures. According to a review from 2020, in the foreseeable future, 19 MW of installed electrolyzer capacity will be connected to biological methanation in Europe [128]. Besides biological methanation taking place above ground, geo-methanation, where CO_2_ and H_2_ are injected into a porous underground, is an appealing option. Depleted gas reservoirs, accounting for 80–90% of the total natural gas underground storage capacity, offer a great potential to serve as the biological methanation reactors of the future, overcoming the limitation of scale [130].

### 3.5. Electro-Microbiology

One example for the application of electro-microbiology are microbial fuel cells (MFC) that have been proposed as means for energy recovery from organic matter during wastewater purification. Taking into account Coulombic loss and voltage efficiency, about 44% of the inherent energy in biodegradable COD can be recovered as electricity, i.e., 1959 kJ·m^−3^, equivalent to 0.54 kWh·m^−3^ [218]. On a global scale, about 360 km^3^·yr^−1^ of municipal wastewater is produced according to a model simulation and historical records [219,220]. Applying an emission factor of 543 g per kWh electricity [221], the total saving potential corresponds to 106 megatonnes CO_2eq_. However, whether MFCs will find their way from labs to application is under debate. In addition to the field of MFCs, it has been proposed that electro-microbiology might considerably contribute to the sector’s microbial production of green chemicals via electro-fermentation and to electro-methanation. The GHG mitigation potential of both areas, i.e., green chemicals and biomethanation, was already assessed above. Given the currently limited state of the art, a forecast of the future impact is almost impossible. A study on the integration of renewable electricity with three emerging bioelectrochemical technologies in a circular bioeconomy scenario predicts a certain potential to improve the biomethane yield, whilst achieving simultaneous biogas production and upgrading, however, with a certain ambiguity [222].

### 3.6. Hydrogenases

Due to its versatility, clean hydrogen is currently enjoying an unprecedented political and business momentum, with the number of policies and projects around the world expanding rapidly [223]. It is predicted that by 2050, hydrogen will meet 12% of the global energy demands according to the International Renewable Energy Agency (IRENA) [224], and the industrial hydrogen demand is expected to increase by 44% until 2030 according to the IEA [225]. The current global hydrogen demand of 75 megatonnes per year is predominantly met with the so-called ‘grey’ hydrogen produced from natural gas. This non-renewable production path is responsible for more than 900 megatonnes of CO_2eq_ [152]. Low-emission alternatives include ‘blue’ hydrogen produced from natural gas combined with carbon capture and storage, and ‘green’ hydrogen produced via water electrolysis using renewable electricity [226]. The third option, biological H_2_ formation utilizing dark fermentation or biophotolysis, is currently less technologically mature. It depends either on the availability of biomass against competing uses or capital-intensive photobioreactors, and is considered more costly compared to green and blue hydrogen [222]. However, a study estimates the current production cost of microalgae-based biohydrogen, ranging from 10 to 20 USD per GJ, depending on regional gas prices [166]. For comparison, the cost of hydrogen production from natural gas ranges from 3.5 to 12 USD per GJ [152]. Even though the authors of the mentioned study concede that cost estimates for biohydrogen might be quite optimistic, it demonstrates that certain biohydrogen technologies are coming close to market readiness. As a first easy implementable option, several studies address the modification of anaerobic digestion toward a two-staged system for the co-production of hydrogen and biogas [227].

### 3.7. Cellulosome

Plant biomass is the most abundant renewable source of carbon accessible to humanity, with an estimated 181.5 gigatonnes produced annually [228]. Currently, a 4.5% share is used with 8.2 gigatonnes from agricultural, grass, and forest land, and about 1.2 gigatonnes stem from agricultural residues [229]. Depending on the source, a cellulose content of 35–55% could be directed toward a sustainable bioeconomy [228]. To gain access to the valuable sugars contained in this recalcitrant biomass, cellulolytic enzymes play a crucial role. Multienzyme complexes, such as cellulosomes, have been described as early as 1983 and have been among the dominant paradigms to utilize the sugars contained in cellulosic biomass [230]. Targeted research by NREL in collaboration with Novozymes and DuPont Industrial Biosciences, the two leading enzyme companies, has proven the dramatic synergistic effect of cellulosomes in combination with free cellulases to break down the recalcitrant biomass fast [184]. Such increased enzymatic activity within cellulose alongside the cheaper cost for enzyme production of <USD 0.11–0.13 per liter ethanol produced brought down the projected cost for a commercial-scale biomass-based ethanol production, and thus cost-effective biofuels [231]. The Billion Ton Bioeconomy Initiative, as described by the U.S. Biomass Research and Development Board, envisions to convert around a billion dry tonnes of biomass into biofuels and bioproducts in the U.S. by 2030 [232]. Such a scenario could avoid 105 megatonnes of CO_2eq_ annually by replacing 9.5% of fossil energy consumption, while boosting the bioeconomy revenue by a factor of 5, to an estimated total of USD 259 billion, and contribute 1.1 million jobs to the U.S. economy [233].

### 3.8. Nitrogenase

The biggest future impact of biological nitrogen fixation is expected from its transfer to cereal crops. Cereals are a primary source of nourishment. For the example of the U.S., three times more corn is used for the production of sweeteners and food starch than what is directly consumed by humans. The FAO estimated the world cereal production in 2022 to be 2777 megatonnes. According to another FAO estimate [234], the total global agricultural consumption of elemental N from synthetic fertilizers reached 113.3 Mt in 2020. The manufacturing and transportation of synthetic N fertilizer was responsible for estimated emissions of 492 megatonnes CO_2eq_ [235]. Considering that around 60% of the global N-fertilizer is applied to cereal crops, the potential saving is 295 megatonnes CO_2eq_. Besides the avoidance of GHG emissions related to fertilizer reduction, there are additional benefits. Typically, only about half of the N applied is taken up by the crop during that growing season [236]. N_2_O, a potent long-lived GHG, is produced by microbes in soils, and emissions are especially high when the N fertilizer is applied at rates exceeding the crop need [237]. The estimated impact of human N fixation on N_2_O emission is 8.0 megatonnes N_2_O-N·yr^−1^, which is equal 1.0 gigatonnes CO_2eq_·yr^−1^ [235]. It has been stressed that cereals capable of fixing their own N would have their supply matched to their N demand, and thus strongly reduce N_2_O emission [238]. However, the aim of creating ‘N-self-fertilizing’ cereals, avoiding the need for chemical fertilizers, still lies ahead [239].

## 4. Conclusions

The potential future impacts of the biotechnological tools discussed in this manuscript are subject to personal interpretation. Our focus was on biological instruments and procedures shaped by nature’s competition for survival. Some powerful instruments, such as genetic and metabolic engineering, synthetic biology, and biocomputing, were not explicitly mentioned, but are expected to play a crucial role in all advancing biotech processes.

Another significant invention that was not listed is photosynthesis, which has already demonstrated its huge potential to reduce atmospheric CO_2_ through assimilation in the biosphere. Photosynthetic approaches will be a key component in addressing climate change, but the vast array of biotech developments and concepts, including artificial leaves, chlorophyll-based photovoltaic cells, genetically modified crops, and microalgae biorefineries, requires further review. According to a high-aiming outlook on the European biorefinery, by 2030, a significant proportion of Europe’s demand for chemicals, energy, materials, and fibers will be met using a biomass feedstock for biorefining technologies, with 30% of the chemical production being biobased and more than 50% for high-value chemicals and polymers. Biofuels will supply 25% of Europe’s transport energy needs, with advanced fuels such as biobased jet fuels becoming increasingly prevalent [240]. Not only Europe, but all industrialized nations share similar ambitions. The U.S. Department of Energy’s Bioenergy Technologies Office (BETO) is also investing in a diverse portfolio of new biomass-derived chemicals and bioproducts as part of a pathway to full decarbonization [241,242], and has set a goal to expand sustainable aviation fuel (SAF) production, with the target of reducing GHG emissions by 50% compared to conventional fuel by 2050. It foresees a scale-up to 35 billion gallons (~132 million m^3^) SAF per year by 2050, with a 3 billion gallons (~11.4 million m^3^) milestone targeted for 2030. As another example, South Korea aims to triple the size of its green biotechnology industry by 2030 as a future growth engine for the economy [243]. These ambitious global plans are supported by significant funding for the R and D and demonstration at the technical scale, and the combination of seed funding and the growing effects of climate crises will drive the implementation of the concepts and processes outlined in this paper.

## Data Availability

No new data were created or analyzed in this study. Data sharing is not applicable to this article.
